# High‐Yield 5‐Hydroxymethylfurfural Synthesis from Crude Sugar Beet Juice in a Biphasic Microreactor

**DOI:** 10.1002/cssc.201901115

**Published:** 2019-08-22

**Authors:** Ria M. Abdilla‐Santes, Wenze Guo, Pieter C. A. Bruijnincx, Jun Yue, Peter J. Deuss, Hero J. Heeres

**Affiliations:** ^1^ Department of Chemical Engineering (ENTEG) University of Groningen 9747 AG Groningen The Netherlands; ^2^ Department of Chemical Engineering University of Brawijaya, MT Haryono 167 Malang 65145 Indonesia; ^3^ Inorganic Chemistry and Catalysis Debye Institute for Nanomaterials Science Utrecht University Universiteitsweg 99 3584 CG Utrecht The Netherlands; ^4^ Organic Chemistry and Catalysis Debye Institute for Nanomaterials Science Utrecht University Universiteitsweg 99 3584 CG Utrecht The Netherlands

**Keywords:** 5-hydroxymethylfurfural, biobased chemicals, microreactors, renewable resources, sucrose

## Abstract

5‐Hydroxymethylfurfural (HMF) is an important biobased platform chemical obtainable in high selectivity by the hydrolysis of fructose (FRC). However, FRC is expensive, making the production of HMF at a competitive market price highly challenging. Here, it is shown that sugar beet thick juice, a crude, sucrose‐rich intermediate in sugar refining, is an excellent feedstock for HMF synthesis. Unprecedented high selectivities and yields of >90 % for HMF were achieved in a biphasic reactor setup at 150 °C using salted diluted thick juice with H_2_SO_4_ as catalyst and 2‐methyltetrahydrofuran as a bioderived extraction solvent. The conversion of glucose, obtained by sucrose inversion, could be limited to <10 mol %, allowing its recovery for further use. Interestingly, purified sucrose led to significantly lower HMF selectivity and yields, showing advantages from both an economic and chemical selectivity perspective. This opens new avenues for more cost‐effective HMF production.

## Graphical abstract

**Thick juice**, a crude, high‐sucrose‐containing extract from sugar beets and an intermediate in sugar refining, is identified as an excellent feedstock for the synthesis of 5‐hydroxymethylfurfural and glucose. High yields are obtained in batch and flow in a biphasic setup by using a salted aqueous phase and 2‐methyltetrahydrofuran as extraction solvent.

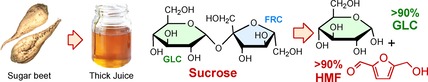

## Introduction

As a response to the anticipated decrease in fossil fuel reserves, fluctuating crude oil prices, and environmental issues related to the use of fossil resources, their replacement with renewable alternatives is receiving much attention. Biomass is an important renewable biobased feedstock, particularly for chemicals and materials production. Here, a major role is assigned to platform chemicals that are readily accessible from biomass and can serve as chemical building block for subsequent divergent transformations into final biobased products.^1^ One identified versatile furanic platform chemical is 5‐hydroxymethylfurfural (HMF).^2^ It is attainable from different carbohydrate sources through dehydration by using cheap mineral acids catalysts such as HCl and H_2_SO_4_ and can be converted into a wide range of commodity chemicals and products.^3^ Different carbohydrate feedstocks have been investigated for HMF synthesis ranging from monomeric carbohydrates, such as glucose (GLC) and fructose (FRC), to polymeric carbohydrates such as starch and cellulose.2a, 4 Of the readily accessible monomeric carbohydrates, FRC has been shown to be the most suitable substrate because it offers the highest HMF selectivity, whereas the considerably cheaper GLC leads to low selectivity.2a, 5 When considering the technoeconomic viability of HMF production on a large scale, the feedstock costs are a major contributor to the overall manufacturing costs. As such, the identification of alternative, abundant, and cheap carbohydrate sources, preferably rich in FRC, is of high interest.^3^

Sucrose (SUC), a disaccharide of FRC and GLC, has been explored for HMF synthesis.^6^ SUC, widely available as table sugar, is cheaper than pure FRC and is produced in large volumes from sugar beet and sugar cane. In 2017, the global sugar production was estimated to be 179.6 million tonnes, with the EU contributing to 10 % of the total amount.^7^ Until 2017, the production of sugar in the EU was strictly regulated by the European Commission.^8^ From 2017 onwards, restrictions were abandoned, which led to increased production levels in the EU and lower SUC prices in the long term.^9^ As such, there is an incentive for sugar beet refiners to explore new markets and possibilities. Meanwhile, the European Commission has targets to replace 30 % of fossil‐based chemicals and materials with biobased versions and to supply 25 % of Europe's transport energy needs by using sustainable advanced biofuels by 2030.^10^ As such, both from a price and legislation perspective, the coproduction of biobased products such as HMF in a sugar beet refinery is highly attractive.

Thick juice is an intermediate product in a typical sugar beet refinery. It is a viscous, clear, yellow liquid rich in SUC (typically 60–70 wt %) with residual water, various salts, organic acids, and minerals (see Table S1 in the Supporting Information for details provided in the literature).^11^ To obtain crystalline SUC, the thick juice is treated in specially designed vacuum pans to remove water and allow crystallization.^12^ Thick juice can be stored (for up to 1 year) and used for the production of sugar after the sugar beet processing campaign. For the coproduction of biobased chemicals in a sugar refinery, the use of thick juice instead of crystalline SUC is expected to be beneficial because the costly crystallization step is avoided. The conversion of thick juice to bioethanol11a, 13 and biohydrogen^14^ through fermentation routes has been successfully demonstrated. However, studies on (chemo‐)catalytic valorization routes of thick juice to platform molecules such as HMF are limited to only one report from our group.11b

We have recently reported a comprehensive kinetic study on the conversion of pure SUC to HMF and levulinic acid (LA) by using H_2_SO_4_ in water.6a The initial step involves rapid hydrolysis of the glycosidic bond between the sugar monomers in SUC to give FRC and GLC. Subsequently, FRC is dehydrated to give HMF in moderate yield. For example, only 22 mol % HMF yield was obtained from an aqueous solution of SUC at 140 °C with H_2_SO_4_ as the catalyst.6a The formation of HMF from GLC was shown to be slow and to occur with a low selectivity,^15^ and it is thought to require an initial isomerization step of GLC to FRC.^16^ The main reason for the low HMF yields from carbohydrates in aqueous systems is the formation of byproducts such as the soluble and insoluble oligomers and polymers known as humins. Additionally, HMF itself is not stable under the prevailing reaction conditions and is easily converted to LA and formic acid (FA). For these reasons, alternative dehydration methodologies have been developed, including biphasic concepts with advanced catalyst systems^17^ and reactor designs^18^ as well as combinations of alternative solvent systems using either monophasic or biphasic liquid–liquid systems with catalysts.2a, 19 Biphasic systems often involve an aqueous phase with the catalyst and an organic phase with a high affinity for HMF. Methyl isobutyl ketone (MIBK) and *n*‐butanol are among the most common organic solvents used and have been shown to lead to reduced byproduct formation by extraction of HMF from the reactive aqueous phase.3b, 20 This effect can be enhanced by the addition of salts such as NaCl to the aqueous phase.^21^ An overview of selected reports on the conversion of SUC to HMF in biphasic systems is provided in Table S2 (in the Supporting Information).

**Figure 1:**
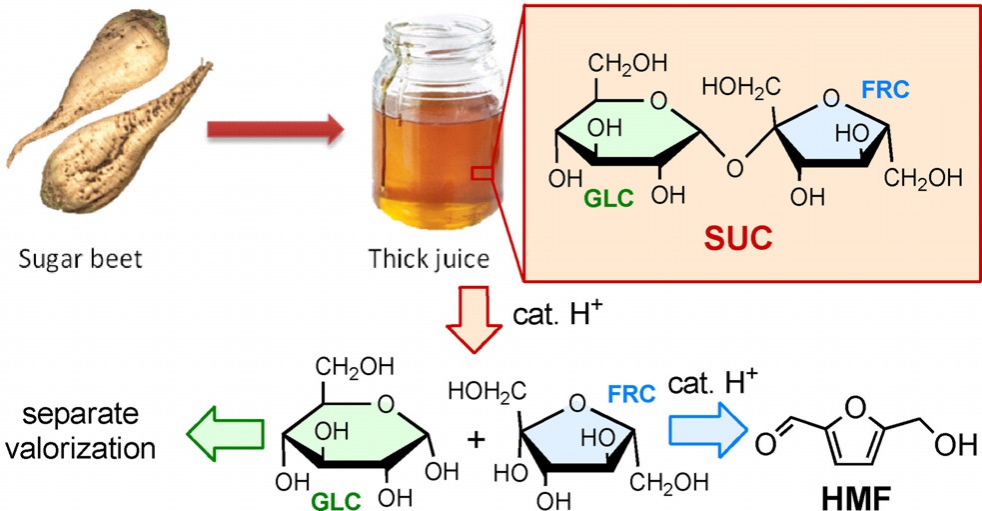
Sugar beet thick juice‐based SUC to HMF and GLC strategy applied in this work.

When using SUC as a source for HMF, two main strategies can be considered: in the first approach, FRC is converted to HMF, and the remaining GLC is in situ isomerized to FRC and subsequently converted to HMF. For this, effective isomerization catalysts are required, and well‐known examples are CrCl_3_ and SnCl_4_.^22^ However, these catalysts are expensive, toxic, and do not always show the desirable compatibility with the dehydration reaction. Additionally, this method typically requires the use of ionic liquids or other alternative, expensive solvents to obtain high HMF yields from GLC. The second approach relies on the conversion of SUC to HMF (from FRC) and GLC. This is possible through careful control of the reaction conditions (Figure 1) by making use of the fact that FRC is significantly more reactive than GLC at temperatures around 140 °C.6a, 23 This then allows for GLC to be separated after the reaction and subsequently processed to other biobased chemicals or isomerized to FRC and then recycled to the reactor.

**Figure 2:**
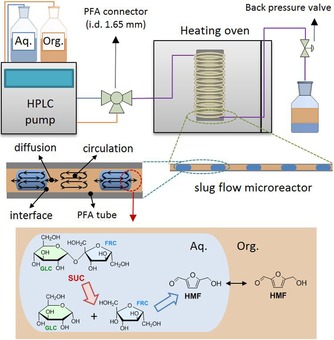
Schematic overview of the continuous slug‐flow microreactor setup used in this study to allow for efficient HMF and GLC synthesis from SUC contained in sugar beet thick juice.

Here, we present a study on the synthesis of HMF and GLC from thick juice in a biphasic liquid–liquid system by using H_2_SO_4_ as the catalyst. Initial experiments were performed in a batch reactor, with or without salt addition. In addition to commonly used MIBK, 2‐methyltetrahydrofuran (MTHF) was used as an extraction solvent, which is considered a green, biobased solvent obtained by hydrogenation of LA or γ‐valerolactone (GVL).^24^ This approach showed that unexpectedly high HMF yields and good yields of coproduced GLC can be achieved from thick juice. Subsequently, the use of a biphasic continuous microreactor operated in the slug‐flow regime was shown to be extremely beneficial for further improving HMF selectivity (Figure 2).^25^ The results were compared with those obtained for pure SUC, clearly showing the beneficial effect of the use of crude thick juice for HMF and GLC production.

## Results and Discussion

### Thick juice and sucrose biphasic reactions in a batch setup

**Figure 3:**
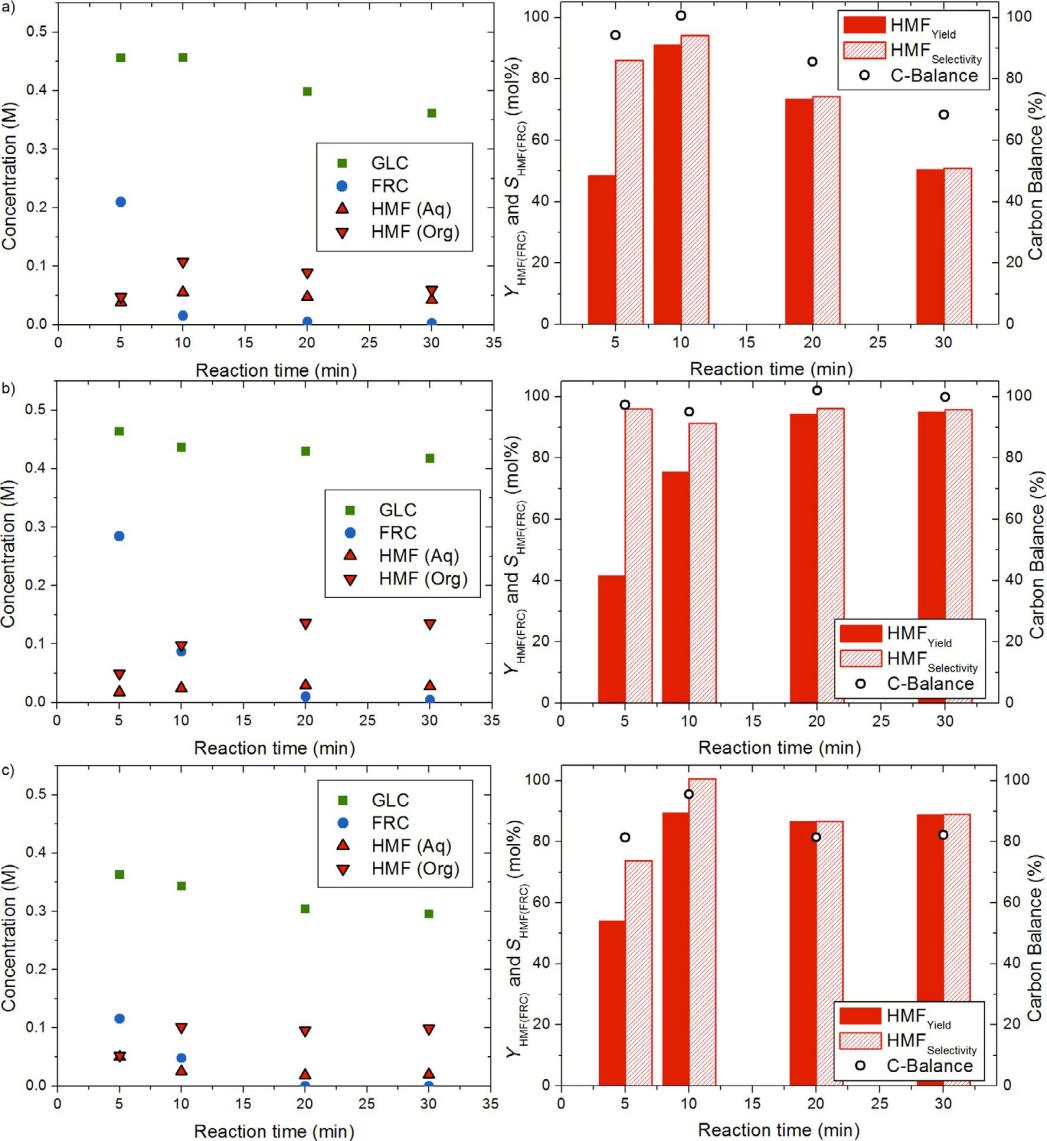
Concentration–time profile (left) and yield, selectivity, and carbon balance (right) of H_2_SO_4_‐catalyzed SUC hydrolysis in a biphasic system with MTHF as the extraction phase: a) thick juice without addition of salt (*C*_SUC(equiv.),0_=0.5 m, pH_aqueous_=0.7 at 25 °C), b) thick juice with added 0.3 g mL^−1^ NaCl (*C*_SUC(equiv.),0_=0.5 m, pH_aqueous_=0.7 at 25 °C), and c) pure SUC with added 0.3 g mL^−1^ NaCl (*C*_SUC(equiv.),0_=0.5 m, pH_aqueous_=0.7 at 25 °C). Reaction conditions: *T*=150 °C, aqueous/organic ratio: 1:4 *v*/*v*.

Initial reactions were performed in batch mode by using stirred glass pressure tubes. To ensure low GLC conversion and to avoid excessive humin formation, the temperature was set to 150 °C and an extraction solvent was used.6a The reactions, monitored at different reaction times, were run at equal H_2_SO_4_ concentrations (0.05 m, pH 1.6) for MTHF (Figure S1 a in the Supporting Information) and MIBK (Figure S2 in the Supporting Information) as well as at a set pH achieved by the careful addition of H_2_SO_4_ (pH 0.7 at 25 °C, Figure 3 a). Because HMF is highly soluble in water, an excess of the organic solvent (4:1, *v*/*v*) was used to ensure high extraction efficiencies.^26^

Because SUC is immediately converted to GLC and FRC at acidic pH, the SUC concentration is not shown in the graphs. At pH 1.6, half of the released FRC was converted after approximately 30 min for both biphasic solvent systems, whereas at pH 0.7 full conversion was achieved within 20 min. A higher HMF selectivity was observed when using MTHF compared with MIBK (82 and 75 %, respectively, after 30 min), which was attributed to the higher partition coefficient of HMF in MTHF/water (*P*=1.9; determined experimentally) compared with HMF in MIBK/water (*P*=1.0), demonstrating the beneficial effect of more extensive removal of HMF from the acid aqueous phase.^17^, ^27^ Low GLC conversion (<10 %) and an overall excellent carbon balance of >90 % was observed at incomplete FRC conversion for both extraction solvents and even for MTHF at pH 0.7. After full FRC conversion was reached, the carbon balance gradually decreased owing to unselective GLC conversion as well as HMF decomposition. This is likely associated with the formation of humin substances, which are not detected by HPLC.

To further improve HMF extraction from the aqueous phase, the effect of NaCl (0.3 g mL^−1^) on HMF production from thick juice was determined in batch at equal H_2_SO_4_ concentrations (MTHF, Figure S1 b; MIBK, Figure S3 in the Supporting Information). The addition of salt led to a nearly two‐fold increase for the partition coefficient of HMF (from 1 to 1.8 for MIBK and from 1.9 to 3.7 for MTHF) by the salting‐out effect, in line with other studies.17a, 17b, 28 This led to significantly higher HMF selectivity (>90 %) at high FRC conversion (>99 %) and low GLC conversion (<10 %). Because the additional of salt also led to a significant pH drop and thus increase in reaction rate,^29^ the reactions with and without salt were also compared at equal pH (MTHF, Figure 3 b). Satisfyingly, this also resulted in a high HMF selectivity (96 %) although at the cost of GLC (17 % conversion) at similar FRC conversion (>99 %, 30 min). A key observation was that the concentrations of HMF did not decrease in these experiments, which is very different compared with previous reports using pure SUC6a as well as from our own comparative experiments (shown below). Indeed, an excellent carbon balance closure (>95 %) was found even at such high FRC conversions. Humin formation was observed in the form of black precipitate and some coloration of the extraction liquid, but to a very minimal extent. Additionally, only traces of LA and FA were detected by HPLC. In these experiments, the latter two were even excluded from the carbon balance because the concentrations were too low for adequate quantification. In addition to NaCl, experiments were performed with Na_2_SO_4_ (Figures S4 and S5 in the Supporting Information). Although the salting‐out effect for salts with double‐charged anions is significantly larger than for salts with single‐charged ions (for NaCl: from 1.8 to 3.5 with MIBK and from 3.7 to 4.4 with MTHF),^30^ this did not lead to improved HMF yield owing to the anion effects on the different reaction rates.17b, 31

To compare the performance of thick juice to purified sucrose, similar biphasic reactions were performed with SUC by using MTHF as the extraction solvent (pH 0.7 Figure 3 c, pH 0.3 Figure S1 c in the Supporting Information). In general, the reactions with pure SUC as the starting material show somewhat higher conversion rates for both FRC and GLC compared with the reactions with thick juice even at equal pH. This is accompanied by a lower selectivity for HMF (87–89 mol % at 20–30 min) compared with thick juice (96 mol % at 20–30 min). Some contribution from GLC to the HMF yield can also not be excluded in this case because GLC conversion is significantly higher (>40 mol % after 30 min). LA and FA are formed (up to 0.04 m, see Figure S6 in the Supporting Information) and the carbon balance (including LA and FA) is significantly lower (82 %) compared with thick juice. This indicates the formation of substantial amounts of unidentified compounds (e.g., soluble humins) as also evident from the increased formation of black precipitate as well as significantly more coloration of the extraction phase. A possible explanation for the observed difference between pure SUC and thick juice must lie with the difference in composition. Besides SUC, thick juice contains salts, organic acids, and other minor components (Table S1 in the Supporting Information).^11^ However, the presence of the salts alone cannot explain the difference, as demonstrated by an experiment of pure SUC in which NaCl was added to the level of those present in thick juice, which led to an overall 10 % lower carbon balance (Figure S7 in the Supporting Information). What (combinations of) components in thick juice cause these improved results for HMF synthesis is the subject of further studies. Here, we focused on optimizing the yields of HMF from thick juice even further.

### Thick juice and sucrose biphasic reactions in a continuous slug‐flow microreactor setup

**Figure 4:**
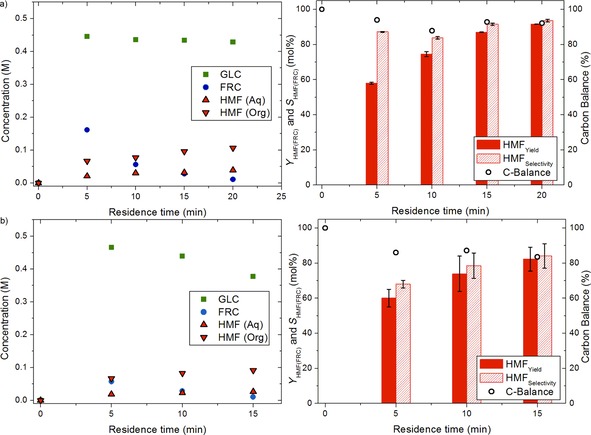
Concentration–time profile (left) and yield, selectivity, and carbon balance (right) of a) thick juice and b) SUC hydrolysis in slug‐flow microreactor in a biphasic system with MTHF in the presence of NaCl (0.1 g mL^−1^) at different residence times obtained by adjusting the flow rate. Solid bar: HMF yield (FRC‐based); shaded bar: HMF selectivity (FRC‐based); circle: carbon balance. Reaction conditions: *C*_SUC(equiv.),0_=0.48 m, *T*=150 °C, aqueous/organic ratio 1:4 *v*/*v*, pH_aqueous_=1.2 (25 °C). Error bars represent average values from three separate experiments.

With the obtained excellent results in hand for the conversion of SUC in thick juice to HMF, the reaction was performed in a continuous slug‐flow microreactor to further improve performance (Figure 2). The use of such a setup leads to improved mass‐transfer characteristics and thus a more efficient extraction of HMF from the aqueous phase as well as improved heat transfer.18b, 32 MTHF was selected as the solvent of choice based on the results obtained in batch (see above). A microreactor made up of a hydrophobic perfluoroalkoxy alkane (PFA) tubing was used, and as such the aqueous phase is present as droplets and the MTHF phase forms the slugs (Figure S8 in the Supporting Information). This was done to prevent significant deposition of humins because these are initially formed in the aqueous phase and thus will not come into contact with the reactor wall. The experimental conditions were similar to the batch system except for the pH, which was set to 1.2, and the use of a lower NaCl concentration (0.1 g mL^−1^ aqueous) instead of 0.3 g mL^−1^ to avoid clogging of the tube as a result of salt precipitation at the reactor outlet. Residence time variations between 5 and 20 min (obtained by adjusting the flow rates) were explored (Figure 4 a, see the Supporting Information for details on the flow rates and residence time determinations). Excellent performance with respect to HMF selectivity was found in the microreactor, showing stable HMF production over extended run times of up to 10 h (Figure S10 and Table S4 in the Supporting Information). HMF yields as high as 91.6 mol % (93.6 mol % selectivity, 97.7 mol % FRC conversion) were obtained at a residence time of 20 min. In addition, GLC conversion was very limited and at most 11.2 mol % at 20 min residence time. A reaction with thick juice without the addition of salt (NaCl) at higher pH was also performed for reference (Figure S9 in the Supporting Information), showing high selectivity but at lower FRC conversion. The use of higher SUC concentration is beneficial for increasing the space–time yield. In our setup, we could run a thick juice solution diluted to 1 m SUC_equiv._. This also led to good HMF selectivity (Table S5 in the Supporting Information) even after 10 h runtimes (Table S4 in the Supporting Information). Operation with less dilute thick juice was not possible owing to operational issues with the feed pump related to the high viscosity of the feed.

Comparison of the results from the batch and flow experiments shows that the conversion of FRC was significantly higher in the microreactor, and particularly the HMF selectivity was improved considerably if no salt was added (Table 1, entries 1–4). High yields could be obtained in both setups although here a higher salt concentration was used in batch (Table 1, entries 5–7). Thus, the use of a microreactor can be beneficial for thick juice conversion compared with a batch reactor. It is interesting to compare the performance of the thick juice in the microreactor with that of pure SUC at the same pH (Figure 4 b). In the microreactor, thick juice performed significantly better than purified SUC. The selectivity of HMF from the reaction with thick juice was higher at all residence times, giving 93.6 mol % at 97.7 mol % FRC conversion compared with only 84 mol % for the reaction with pure SUC at the same FRC conversion. In addition, the GLC conversion of 22.7 mol % was far higher in experiments with pure SUC compared with experiments (at 15 min residence time) with thick juice (10.1 mol %), which is not advantageous considering that GLC conversion does not lead to HMF but mostly to humins. Consequently, the carbon balance for SUC is worse than for thick juice, indicating that SUC gives higher amounts of unidentified compounds such as oligomeric and polymeric humin‐type products. Also, some precipitation of such humins causes disturbance of the flow patterns and eventually leads to blockage, leading to lower reproducibility of the results from this reaction, as was evident from the larger standard deviations from multiple experiments. These differences between the use of thick juice and pure SUC in the microreactor are in line with the obtained batch data discussed above.

**Table 1:** Comparison of data for thick juice to HMF reaction in batch and continuous setups.^[a]^

Entry	Setup	*t* ^[b]^ [min]	pH	*C*_NaCl_ [mg mL^−1^]	*X*_FRC_^[c]^ [mol %]	*S*_HMF_^[c, d]^ [mol %]
1	batch	15	1.6	–	24.1	76.7
2	cont.	15	1.6	–	43.9	84.1
3	batch	30	1.6	–	51.2	81.7
4	cont.	30	1.6	–	65.8	87.4
5	batch	20	0.7	0.3	99.2	95.7
6	cont.	15	1.2	0.1	94.4	91.5
7	cont.	20	1.2	0.1	97.7	93.6

[a] Reactions performed at *T*=150 °C, H_2_O/MTHF ratio: 1:4 *v*/*v*, H_2_SO_4_ as acid catalyst. [b] Batch/residence time. [c] Determined by HPLC. [d] Based on FRC conversion.

### Comparison to literature data for HMF formation

An overview of literature data on the conversion of FRC (and SUC) to HMF in biphasic liquid–liquid systems and a comparison with the data obtained in this study by using thick juice is given in Table 2. Although direct comparison is somewhat skewed owing to variations in process conditions, HMF yields and selectivities based on converted FRC found in this study (batch and continuous) are significantly higher than those reported for pure carbohydrates. For pure SUC and FRC, previous reports typically show 70–75 % selectivity for HMF at similar conditions, something that was also observed in our batch experiments for pure SUC. By using pure SUC in the continuous slug‐flow microreactor and in the presence of an extractive solvent and 0.1 g mL^−1^ NaCl, this could be improved to 84 %, which is already a significant increase over previous reports. A further increase in HMF selectivity to over 90 % was reached by using thick juice as a crude and significantly cheaper SUC feedstock. This is a 15 % higher selectivity compared with previous reports and by far the best achieved thus far in an aqueous solvent system. Better selectivities have previously only been achieved from FRC by using alternative solvent systems such as ionic liquids, which provide significant challenges in the recovery of HMF, solvent, and unconverted carbohydrate.

**Table 2:** Selected examples of HMF yields and selectivities in biphasic liquid–liquid systems in batch and continuous setups with MIBK and MTHF extraction solvents.^[a]^

Entry	Feed	Setup	Conditions	*X*_FRC_ [mol %]	*S*_HMF_ [mol %]	Ref.
1	FRC	batch	aqueous/MIBK 1:4, 150 °C, 30 min	23	43.5	33
2	FRC	batch	aqueous/MIBK 1:4, 160 °C, 2 h	96.8	76	33
3	FRC/GLC^[b]^	cont.	aqueous/MIBK 1:4, 140 °C, 2 h, 0.05 m H_2_SO_4_	94.4	72	23
4	FRC	batch	aqueous/MIBK 1:4, 150 °C, 45 min, 100 g L^−1^ H_3_BO_4_, 50 g L^−1^ NaCl	70	65.7	34
5	SUC	batch	aqueous/MIBK 1:4, 150 °C, 2 h, 100 g L^−1^ H_3_BO_4_, 50 g L^−1^ NaCl	–	70	34
6	FRC	cont.	aqueous/MIBK 1:3, 140 °C, 15 min, 0.25 m HCl	–	74^[c]^	35
7	thick juice	batch	aqueous/MTHF 1:4, 150 °C, 20 min, pH 0.6, 0.3 g mL^−1^ NaCl	99.2	95.7	this work
8	thick juice	cont.	aqueous/MTHF 1:4, 150 °C, 20 min, pH 1.2, 0.1 g mL^−1^ NaCl	97.7	93.6	this work

[a] HMF selectivity shown in the table was calculated based on FRC. [b] 1:1 mixture. [c] Isolated yield.

## Conclusions

Thick juice has significant potential as feedstock for the synthesis of 5‐hydroxymethylfurfural (HMF), as shown here by an aqueous biphasic reaction system using methyl isobutyl ketone (MIBK) or 2‐methyltetrahydrofuran (MTHF) as the extraction solvent in batch and continuous setups. When using this crude sucrose (SUC)‐rich feedstock, excellent selectivities for HMF were achieved in both setups, which surpass all previous reports in aqueous media. The best results in batch were obtained with MTHF in the presence of NaCl, giving 96 mol % selectivity of HMF at near quantitative fructose (FRC) conversion after 15 min reaction time with 14 % glucose (GLC) conversion or 89 % selectivity at higher pH with limited GLC conversion (3.9 mol %) after 30 min reaction time. The use of a continuous microreactor led to a similar HMF selectivity even at lower NaCl concentrations. These high selectivities were achieved by using sulfuric acid as a cheap catalyst and sodium chloride as the sole additive to increase the extraction efficiency. To illustrate the improvement from the use of thick juice further, results with thick juice were compared with those for pure SUC, reaching only 84 mol % HMF selectivity in the microreactor setup. It is highly unusual that feedstock impurities have such a dramatic positive effect on reaction performance, but in this case, it seems that the use of the cheaper crude feedstock offers a significant advantage. Nevertheless, the exact nature that underlies this positive effect is difficult to determine because thick juice is a highly complex mixture of many different components. Although certain salts and organic acids could be beneficial and explain the excellent results for thick juice, further detailed studies are required to investigate this further. Overall, this study implies that thick juice is a very attractive feedstock for HMF synthesis. It is expected to be significantly cheaper than refined SUC and as such will have a positive effect on the technoeconomic viability of HMF production. In addition, the current study showed that low GLC conversions are possible by proper tuning of the reaction conditions, making use of the fact that FRC is by far more reactive than GLC. This is of high relevance because GLC is known to be far less selective for HMF synthesis than FRC. As such, the unconverted GLC, present in the aqueous phase after reaction, may either be isomerized to FRC and recycled to the reactor or converted separately either by chemo‐ or biocatalytic conversions to biobased products (e.g., lactic acid, sorbitol, succinic acid, etc.).

## Experimental Section

### Materials

Thick juice was kindly provided by Suiker Unie‐Royal Cosun. (62 wt % SUC determined by polarimeter and mild hydrolysis by using 5 mm H_2_SO_4_ at 100 °C followed by quantification of FRC and GLC by HPLC), H_2_SO_4_ (96–98 wt %), SUC (≥99 wt %), GLC (≥99.5 wt %), FRC (99 wt %), NaCl, Na_2_SO_4_, MTHF, and FA were obtained from Sigma–Aldrich (Steinheim, Germany). MIBK was acquired from Merck Millipore (Darmstadt, Germany). LA was purchased from Alfa Aesar. All chemicals were used without further purification. For all experiments, Milli‐Q water was used to prepare the solutions.

### Analytical techniques

The SUC content in the thick juice feed was determined by using polarimetry (Schmidt and Haensch, Polatronic MH8). A series of SUC solutions of known concentrations were prepared and the observed rotation (*α*_obs_) of each solution was determined. Measurements were performed at a wavelength (*λ*) of 589.44 nm and in a cell with a length of 100 mm. These data were used to calculate the specific rotation of SUC ([*α*]; see the Supporting Information for details). Afterwards, a solution of thick juice with a known dilution factor was measured and used to calculate the SUC concentration in the thick juice feed. HPLC was used to determine the composition of the aqueous phases after reaction. The instrument consisted of an Agilent 1200 pump, a Bio‐Rad organic acid column (Aminex HPX‐87H), a Waters 410 differential refractive index detector, and a UV detector. The HPLC column was operated at 60 °C, and aqueous H_2_SO_4_ (5 mm) was used as the mobile phase with a flow rate of 0.55 mL min^−1^. The injection volume of the sample was set at 5 μL. Concentrations of compounds in the product mixture were determined by using calibration curves obtained by analyzing standard solutions of known compounds with known concentrations. GC [model: Finnigan, Trace GC Ultra, equipped with flame ionization detector (FID) and Stabilwax‐DA column 30 m×0.32 mm inner diameter and film thickness of 1 μm] was used to determine the composition of the organic phases after reaction. The carrier gas was helium with 2.2 mL min^−1^ flow rate, and the split ratio was set at 50:1. The injector temperature was set at 260 °C. The oven temperature was kept at 40 °C for 5 min, then increased to 240 °C at a rate of 15 °C min^−1^, and then held at 240 °C for 10 min.

### HMF formation in batch experiments

Reactions were performed in Ace pressure tubes (bushing type, front seal, and volume≈9 mL) with a length of 10.2 cm and an outer diameter of 19 mm, equipped with a magnetic stirring bar. The tubes were filled with a 1:4 *v*/*v* ratio of water and an organic solvent (MIBK or MTHF). The water phase contained the appropriate amount of thick juice or SUC and H_2_SO_4_ (0.5 and 0.05 m, respectively). For some experiments with salt (NaCl or Na_2_SO_4_), 0.3 g salt was added per mL of the aqueous phase (containing thick juice or SUC) prior to mixing with the organic phase. As such, the initial concentration of SUC (0.44 m) and catalyst (0.044 m) in the aqueous phase were slightly different. Otherwise the pH value was set by careful addition of H_2_SO_4_ to a salty solution of thick juice or SUC. After filling, the tubes were closed and submerged in a temperature‐controlled heating bath (*T*=150 °C). During the reaction, the mixture was stirred at 500 rpm. At various reaction times, a tube was taken out and quickly immersed in cold water to stop the reaction. The two‐phase reaction mixture was then subjected to centrifugation (HeraeusTM, MegafugeTM 40, 4500 rpm for 10 min), and both phases were separated and collected. Aliquots of the aqueous phase and the organic phase were withdrawn, filtered when necessary [0.45 μm polytetrafluoroethylene (PTFE) filter], and analyzed by HPLC and GC‐FID, respectively.

### HMF formation in continuous microreactor experiments

Reactions were performed in a perfluoroalkoxy alkane (PFA) tube with an internal diameter of 1.651 mm and a total length of 4.5 m. To improve heat transfer, the tube was coiled around a cylindrical shaped aluminum block (50 mm diameter), which was placed inside a temperature‐controlled oven (*T*=150 °C). A schematic representation of the setup is depicted in Figure 2. The aqueous and organic solutions were introduced into the reactor by HPLC pumps (Agilent 1100 with flow rate range of 0–5 mL min^−1^). The aqueous feed contained the appropriate amount of thick juice (0.5 m SUC equiv.) and H_2_SO_4_ (0.05 m). For experiments with NaCl (0.1 g mL^−1^), the initial concentrations of SUC and H_2_SO_4_ in the aqueous phase were 0.48 and 0.048 m, respectively. An aqueous/organic solvent phase ratio of 1:4 was applied. Prior to entering the heating oven, the two liquid phases were combined in a Y‐type connector to create a slug‐flow. Owing to the hydrophobic nature of the tubing, the aqueous phase formed droplets separated by a continuous organic phase. A back‐pressure valve was placed at the outlet of the reactor to adjust the pressure to 8–10 bar. Residence times were set by adjusting the flow rates of the HPLC pump (see Table S7 in the Supporting Information). The reactor typically reached a steady state after approximately two times the residence time. Samples were collected from the outlet, and the two‐phase mixtures were subjected to centrifugation (HeraeusTM, MegafugeTM 40, 4500 rpm for 10 min). Afterwards, both phases were separated, and aliquots of aqueous phase and organic phase were withdrawn, filtered when necessary (0.45 μm PTFE filter), and then analyzed by HPLC and GC‐FID, respectively.

### Determination of yield and conversion

At the typical reaction conditions used in this study (*T*>100 °C), SUC is immediately converted (inverted) into FRC and GLC in the initial stage of the reactions.6a As such, the initial concentrations of the individual sugars were set equal to the initial concentration of SUC in the aqueous phase. For the biphasic reaction, it is known that the solubility of FRC and GLC in the organic phase is very low and therefore assumed negligible for conversion and yield calculations. However, a small amount of organic phase can dissolve into the aqueous phase and vice versa, resulting in a volume changes of both phases (*V*_initial_≠*V*_final_). To compensate for changes in the volumes of the organic phase and aqueous phase after reaction, a factor *R* was applied, which is defined as the ratio of the final volume to the initial volume. *R* was estimated by the software package Aspen (see Table S6 in the Supporting Information). This factor *R* is also used in the conversion and yield calculations. The conversion of FRC and GLC including *R* are given in Equations (1), (2), respectively:

(1) $$X_{FRC}=\frac{N_{FRC,0}-N_{FRC}}{N_{FRC,0}}=\frac{V_{aq}C_{FRC,aq,0}-V_{aq}R_{aq}C_{FRC,aq}}{V_{aq}C_{FRC,aq,0}}$$

(2) $$X_{GLC}=\frac{N_{GLC,0}-N_{GLC}}{N_{GLC,0}}=\frac{V_{aq}C_{GLC,aq,0}-V_{aq}R_{aq}C_{GLC,aq}}{V_{aq}C_{GLC,aq,0}}$$

Unlike the sugars, HMF is present in both the aqueous and organic phase after reaction. The yield of HMF on a SUC basis is given in Equation (3):

(3) $$Y_{{HMF}_{\left(SUC\right)}}=\frac{N_{HMF,tot}}{2N_{SUC,0}}=\frac{V_{org}R_{org}C_{HMF,org}+V_{aq}R_{aq}C_{HMF,aq}}{2V_{aq}C_{SUC,0}}$$

Because the conversion of GLC is very low (which in this work was intentional), the yield of HMF can also be expressed through FRC only (*Y*_HMF(FRC)_), see Equation (4):

(4) $$Y_{{HMF}_{\left(FRC\right)}}=\frac{N_{HMF,tot}}{N_{FRC,0}}=\frac{V_{org}R_{org}C_{HMF,org}+V_{aq}R_{aq}C_{HMF,aq}}{V_{aq}C_{FRC,0}}$$

Similarly, the HMF selectivity can be expressed on a SUC [*S*_HMF(SUC)_, Eq. (5)] or FRC basis [*S*_HMF(FRC)_, Eq. (6)]:

(5) $$S_{{HMF}_{\left(SUC\right)}}=\frac{Y_{{HMF}_{\left(SUC\right)}}}{{X_{FRC}+X}_{GLC}}$$

(6) $$S_{{HMF}_{\left(FRC\right)}}=\frac{Y_{{HMF}_{\left(FRC\right)}}}{X_{FRC}}$$

The carbon balance closure (CBC) is defined as total moles of carbon in HPLC‐detectable compounds (FRC, GLC, HMF, and in some cases FA and LA) at a certain reaction time or residence time divided by the moles of carbon in the feed [Eq. (7)]:

(7) $$CBC=\frac{\mathrm{mol}\;C\;\mathrm{in}\;\mathrm{HPLC}\;\mathrm{detectable}\;\mathrm{compounds}}{\mathrm{mol}\;C\;\mathrm{in}\;\mathrm{the}\;\mathrm{feed}}\times 100\;\%$$

The partition coefficient (*P*) is defined as the ratio of the concentration of a component in the organic phase to the concentration of the component in the aqueous phase. As an example, the partition coefficient of HMF is given in Equation (8):

(8) $$P_{HMF}=\frac{C_{HMF,org}}{C_{HMF,aq}}$$

## Conflict of interest

*The authors declare no conflict of interest*.

## Supporting information

As a service to our authors and readers, this journal provides supporting information supplied by the authors. Such materials are peer reviewed and may be re‐organized for online delivery, but are not copy‐edited or typeset. Technical support issues arising from supporting information (other than missing files) should be addressed to the authors.

Supplementary
