# Supplementary material for: High‐Yield 5‐Hydroxymethylfurfural Synthesis from Crude Sugar Beet Juice in a Biphasic Microreactor
Source: ChemSusChem. 2019 Aug 22;12(18):4304–12. doi: 10.1002/cssc.201901115 (PMC6790971; doi:10.1002/cssc.201901115)
Supplement: Supplementary file 1 — Supplementary [file CSSC-12-4304-s001.pdf]

CHEMISTRY & SUSTAINABILITY
CHEMSUSCHEM
ENERGY & MATERIALS

Supporting Information

High-Yield 5-Hydroxymethylfurfural Synthesis from Crude Sugar Beet Juice in a Biphasic Microreactor

Ria M. Abdilla-Santes+,[a, b] Wenze Guo+,[a] Pieter C. A. Bruijnincx,[c, d] Jun Yue,[a] Peter J. Deuss,*[a] and Hero J. Heeres*[a]

cssc_201901115_sm_miscellaneous_information.pdf

Table S1. Thick juice compositions reported in literature

	Tan et al. (2015)		van Zandvoort (2015)
Lactic acid (mg/L)	13586 ± 545.9	Zn (wt%)	< 0.0002
Acetic acid (mg/L)	2176 ± 117.4	Al (wt%)	< 0.0002
Sucrose (g/L)	640.3 ± 23.8	Mn (wt%)	< 0.0002
Glucose (g/L)	87.9 ± 6.4	Fe (wt%)	< 0.0002
Fructose (g/L)	-	K (wt%)	0.6 – 0.8
NH_4^+ (mg/L)	104.6 ± 3.8	Na (wt%)	0.1
Ca^{2+} (mg/L)	1568 ± 73.5	Malic acid (wt%)	0.02 – 0.1
Mg^{2+} (mg/L)	110.6 ± 7.4	Lactic acid (wt%)	0.2 – 0.5
K^+ (mg/L)	5432 ± 63.6	Acetic acid (wt%)	0.1
		Pyrrolidone carboxylic acid (wt%)	0.2
		Citric acid (wt%)	0.02 – 0.1
		Sucrose (wt%)	60 – 70

Table S2. Selected examples of HMF synthesis from sucrose (SUC) in biphasic systems

No.	SUC amount	Cat.	Cat. amount	Time	Solvent(s)	T (^{o}C)	HMF yield[a] (mol%)	Ref.
1	30 mg	Al-TUD-1	20 mg	2-6 h	H_2O:Toluene 0.3:0.7(v/v)	170	17	[1]
2	10 wt%	HCl	pH 1	5 min	H_2O:DMSO 4:6 (w/w) and MIBK:2-BuOH 7:3 (w/w) with aq:org of 1:2 (w/w)	170	50	[2]
3	10 wt%	none	-	4.5 h	H_2O:DMSO 3:7 (w/w) and DCM with aq:org of 1:1 (w/w)	170	51	[2]
4	3.5 g	H-mordenite Si/Al = 11)	1 g	1 h	H_2O:MIBK 1:5 (v/v)	165	28	[3]
5	10 wt%	H_2SO_4	0.1 N	10 min	H_2O:n-BuOH 1:1 (w/w)	150	30	[4]
6	n.a.	SPC-108	In fixed bed	12 h	H_2O:MIBK 1:3 (v/v)	78	41	[5]
7	23 wt%	TiO_2	In fixed bed	3 min	H_2O:n-BuOH 1:3 (w/w)	180	16	[6]
8	120 g/L ILs	$CrCl_3$	0.04 M	4 h	[BMIM]Cl:MIBK 3:7 (v/v)	100	100	[7]
9	5 wt%	Zr(O)Cl_2	10 mol%	5 min	H_2O:MIBK 1:1 (v/v)	120	39	[8]
	100 mg	ZrP	50 mg	2 h	NaCl-H_2O:Diglyme 1:3 v/v	180	53	[9]
10	2.5 mmol	$InCl_3$	50mM	2h	NaCl-H_2O:THF 1:3 v/v	200	52	[10]
11	0.75 mmol	$ZnCl_2$/HCl	0.2 mmol (27 mol%)	1 h	NaCl-H_2O:THF 1:10 v/v	180	65.6	[11]
12	200 mg	$SnCl_4$	10 mol%	4h	EMIMBr:glycol dimethyl ether 250 mg:2 mL	100	65.7	[12]

[a] Yields are based on monosaccharide concentration;

Specific rotation of SUC

The specific rotation of SUC ([α]) is determined using the following equation:

$$[\alpha]_D^{25} = \frac{\alpha_{obs}}{c\ x\ l} \qquad \text{(S1)}$$

Where:

$[\alpha]_{\mathrm{D}}^{25}$ = specific rotation of the compound at 25 ^{o}C using the D-line of the sodium lamp (λ=589.3 nm)

α_{obs} = observed optical rotation

c = the concentration of the solution in grams per milliliter

l = the length of the cell tube in decimeters

Volume changes in biphasic system

Over the course of reaction, small amount of organic solvent will be soluble in the aqueous phase (and *vice versa*) and consequently change the volume of both phases. This will affect the yield calculations and therefore corrections need to be made regarding the solvents. The changes in volume can be modeled using computer program Aspen (Aspen V7.3 NRTL-RK) and the results can be seen in Table S3. As samples are both taken and analyzed at room temperature, the volume change simulation is done at 20 ^{o}C.

Table S3. Modelled volume changes of aqueous: organic mixtures at 20° C estimated by Aspen

Water – MIBK mixture						
Ratio	V_{aq} (L)		R_{aq}	V_{org} (L)		R_{org}
(Aq:Org)	Initial	Final		Initial	Final	
1:4	100	99.22	0.9922	400	400.78	1.0020
1:2	100	99.48	0.9948	200	199.13	0.9957
1:1	100	100.99	1.0099	100	98.30	0.9830
2:1	200	203.49	1.0175	100	95.77	0.9577
4:1	400	408.48	1.0212	100	90.70	0.9070
For Water – MTHF mixture						
Ratio	V_{aq} (L)		R_{aq}	V_{org} (L)		R_{org}
(Aq:Org)	Initial	Final		Initial	Final	
1:4	100	98.246	0.98246	400	401.754	1.004385
1:2	100	99.123	0.99123	200	200.877	1.004385
1:1	100	99.561	0.99561	100	100.439	1.004385
2:1	200	199.562	0.99781	100	100.439	1.004385
4:1	400	399.562	0.99890	100	100.439	1.004385

$$R_{(aq\ or\ org)} = \frac{Final\ volume}{Initial\ volume} \tag{S2}$$

Thick juice reactions in biphasic system with MeTHF (Equal H_2SO_4 concentration)

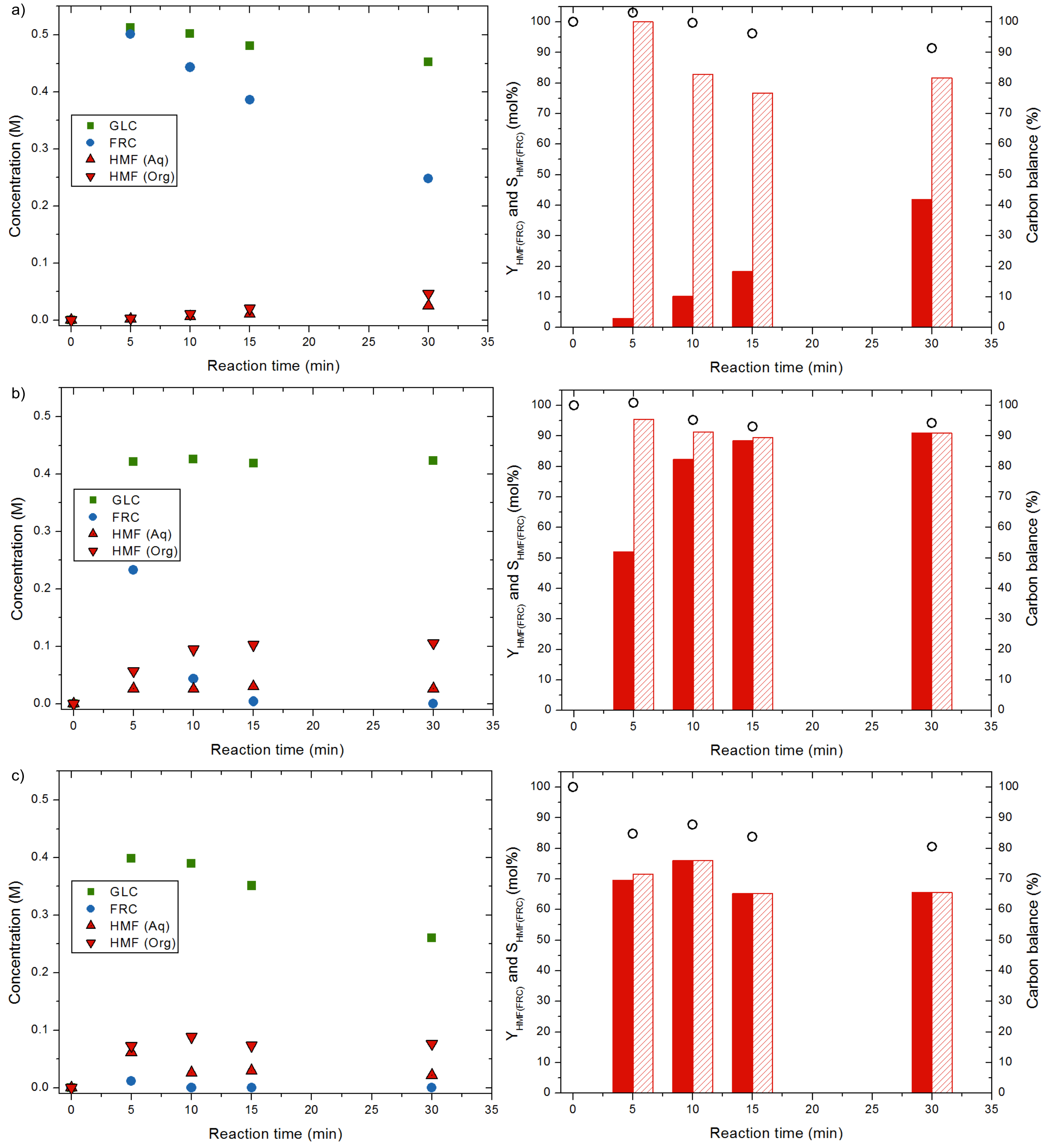

Figure S1. Concentration-time profile (left) and yield, selectivity and carbon balance (right) of SUC hydrolysis in biphasic system with MTHF as extraction phase and a) thick juice without addition of salt ($C_{SUC(equivalent),0}$ = 0.5 M, C_{H2SO4} = 0.05 M, $pH_{aqueous}$ = 1.6 at 25 °C), b) thick juice with added 0.3 g/mL NaCl ($C_{SUC(equivalent),0}$ = 0.44 M, C_{H2SO4} = 0.044 M, $pH_{aqueous}$ = 0.75 at 25 °C) and c) pure sucrose with added 0.3 g/mL NaCl ($C_{SUC(equivalent),0}$ = 0.44 M, C_{H2SO4} = 0.044 M, $pH_{aqueous}$ = 0.3 at 25 °C). Solid bar: HMF yield (FRC based), shaded bar: HMF selectivity (FRC based), circle: carbon balance. Reaction conditions:, T = 150 °C, 1:4 v/v aqueous : organic ratio.

Thick juice reactions in biphasic system with MIBK

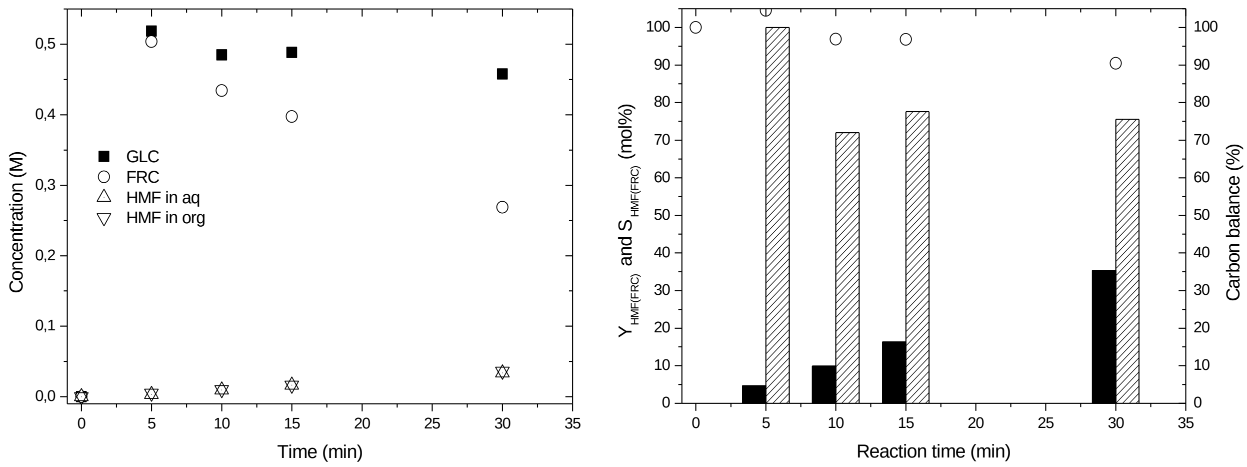

Figure S2. Concentration-time profile (left) and yield, selectivity and carbon balance (right) of thick juice hydrolysis in biphasic system MIBK as extraction solvent. Black bar: HMF yield (FRC based), shaded bar: HMF selectivity (FRC based), circle: carbon balance. Reaction conditions: $C_{SUC(equivalent),0}$ = 0.5 M, T = 150 °C, $C_{H_2SO_4}$ = 0.05 M, 1 : 4 v/v aqueous : organic ratio. $pH_{aqueous}$ = 1.6 (at 25 °C)

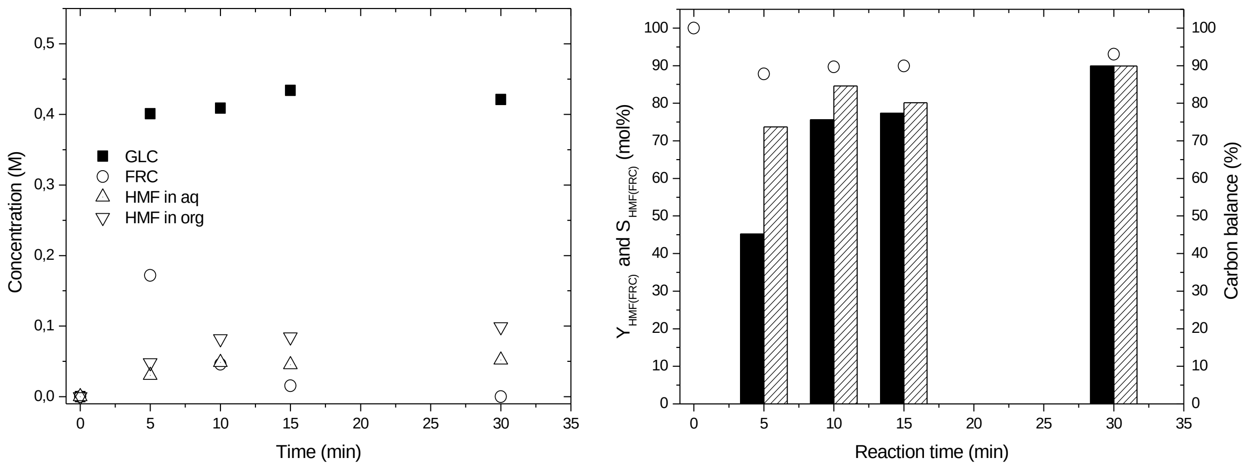

Figure S3. Concentration-time profile (left) and yield, selectivity and carbon balance (right) of thick juice hydrolysis in biphasic system MIBK as extraction solvent and NaCl (0.3 g/mL aqueous). Black bar: HMF yield (FRC based), shaded bar: HMF selectivity (FRC based), circle: carbon balance. Reaction conditions: $C_{SUC(equivalent),0}$ = 0.44 M, T = 150 °C, $C_{H_2SO_4}$ = 0.044 M, 1 : 4 v/v aqueous : organic ratio. $pH_{aqueous}$ = 0.7 (at 25 °C)

Thick juice reactions in biphasic system with addition of Na_2SO_4

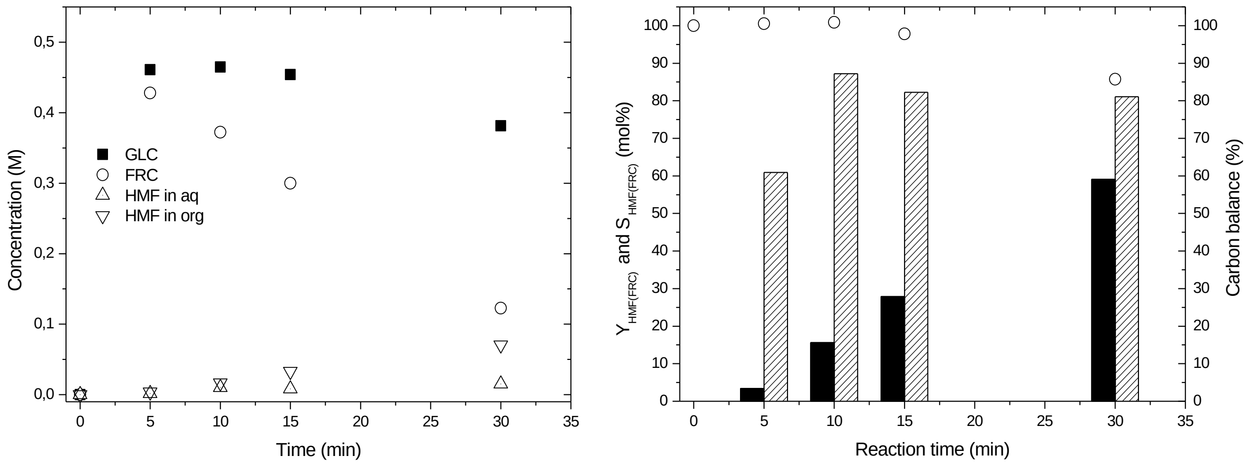

Figure S4. Concentration-time profile (left) and yield, selectivity and carbon balance (right) of thick juice hydrolysis in biphasic system with MIBK as extraction solvent and Na_2SO_4 (0.3 g/mL aqueous). Black bar: HMF yield (FRC based), shaded bar: HMF selectivity (FRC based) only, circle: carbon balance. Reaction conditions: $C_{SUC(equivalent),0}$ = 0.45 M, T = 150 °C, 500 rpm, $C_{H_2SO_4}$ = 0.045 M and 1 : 4 v/v aqueous : organic ratio. $pH_{aqueous}$ = 2.3 (at 25 °C).

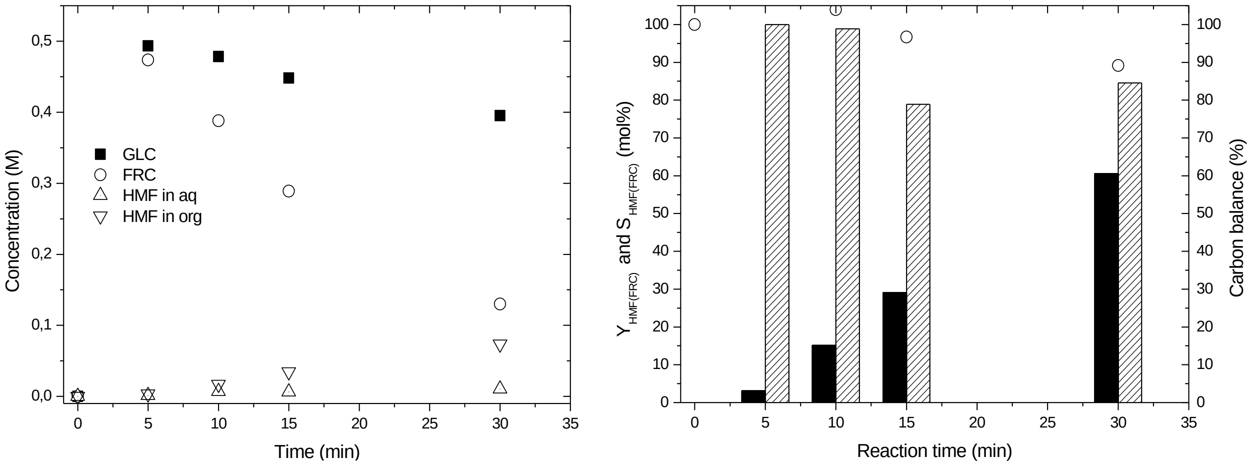

Figure S5. Concentration-time profile (left) and yield, selectivity and carbon balance (right) of thick juice hydrolysis in biphasic system with MTHF as extraction solvent and Na_2SO_4 (0.3 g/mL aqueous). Black bar: HMF yield (FRC based), shaded bar: HMF selectivity (FRC based) only, circle: carbon balance. Reaction conditions: $C_{SUC(equivalent),0}$ = 0.45 M, T = 150 °C, 500 rpm, $C_{H_2SO_4}$ = 0.045 M and 1 : 4 v/v aqueous : organic ratio. $pH_{aqueous}$ = 2.3 (at 25 °C).

Formic acid (FA) and levulinic acid (LA) formation in SUC reaction in biphasic system with addition of NaCl

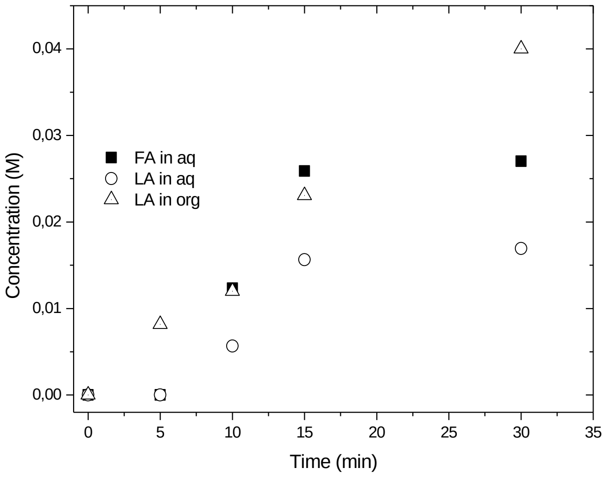

Figure S6. Concentration-time profile for FA and LA obtained from SUC hydrolysis in biphasic system with MTHF extraction solvents and addition of NaCl (0.3 g/mL aqueous). Reaction condition: $C_{SUC(equivalent),0}$ = 0.44 M, T = 150 °C, 500 rpm, $C_{H_2SO_4}$ = 0.044 M and 1 : 4 v/v aqueous : organic ratio. pH aqueous = 0.3 (25 °C)

FA was only detectable in the aqueous phase. For the purpose of carbon balance calculation, the concentration of FA in the organic phase (in this case MTHF) was estimated using the partition/distribution coefficient value of FA in MTHF-water system found in the literature – which is ranging from 1.41 – 1.71 at room temperature [13, 14]. A partition coefficient value of 1.56 (an average value from literature data) is then use to estimate FA concentration in this reaction.

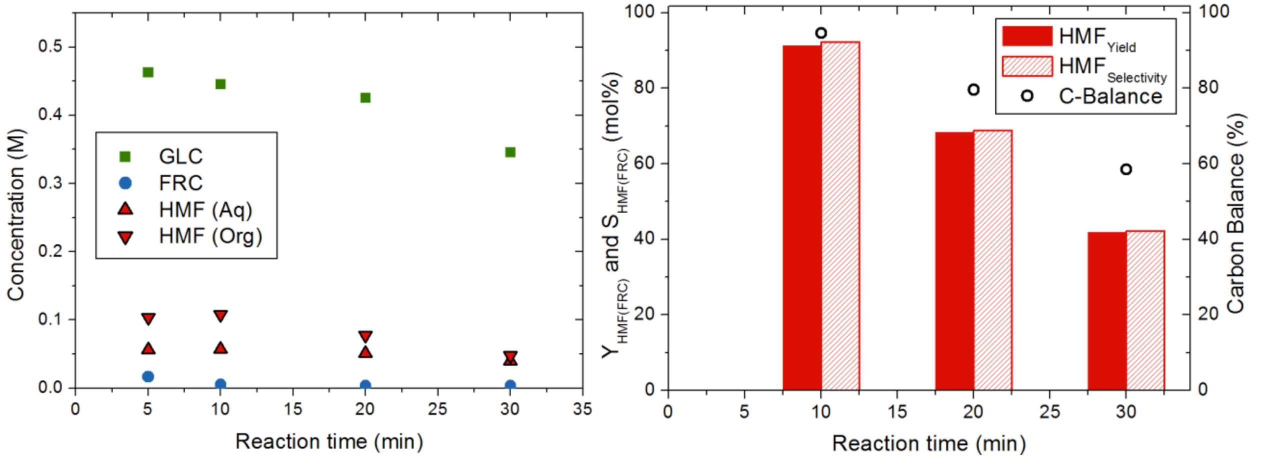

Figure S7. Concentration-time profile (left) and yield, selectivity and carbon balance (right) of SUC hydrolysis in biphasic system MTHF as extraction solvent and NaCl (0.003 g/mL aqueous). Reaction conditions: $C_{SUC(equivalent),0}$ = 0.5 M, T = 150 °C, 1 : 4 v/v aqueous : organic ratio. $pH_{aqueous}$ = 0.7 (at 25 °C)

Slug flow microreactor

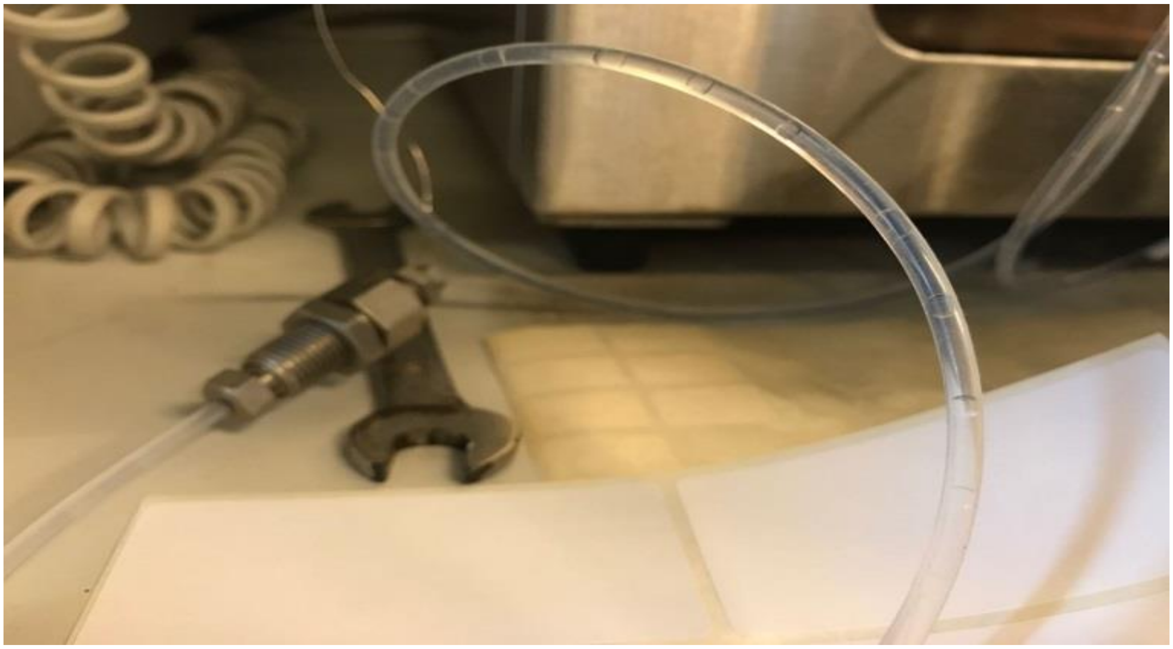

Figure S8. Photograph of the obtained slug flow of the aqueous phase diluted thick juice (smaller droplets) 0.5 M sucrose equivalents and the MeTHF organic phase (larger slugs) ratio 1 : 4 (before reaction).

Thick juice reaction in continuous slug-flow microreactor without the addition of salt

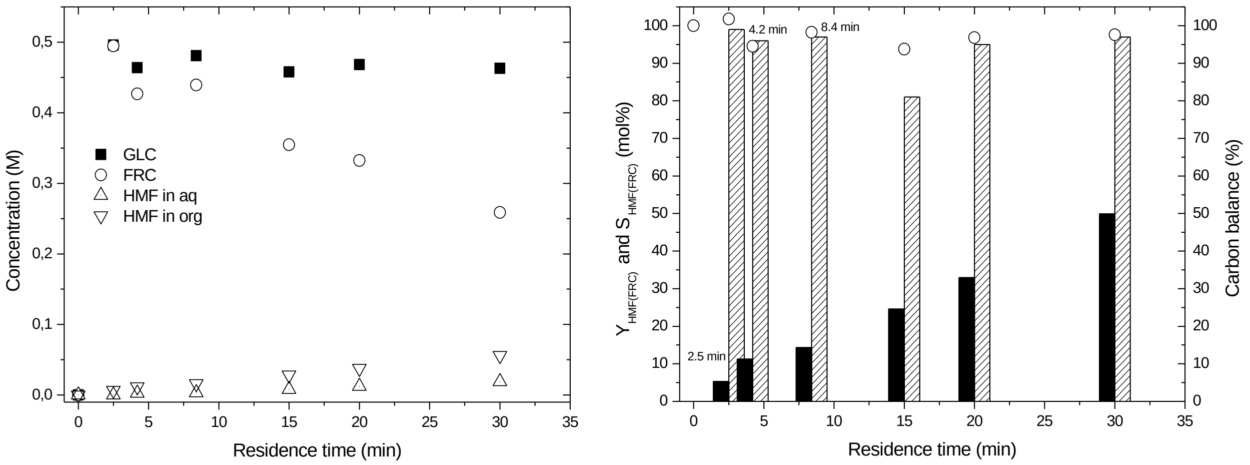

Figure S9. Concentration-time profile (left) and yield, selectivity and carbon balance (right) of thick juice hydrolysis in slug flow microreactor in biphasic system with MTHF extraction solvents, without addition of NaCl over various residence times. Black bar: HMF yield (FRC based), shaded bar: HMF selectivity (FRC based), circle: carbon balance. Reaction condition: $C_{SUC(equivalent),0}$ = 0.48 M, T = 150 ^oC, $C_{H_2SO_4}$ = 0.048 M, 1:4 v/v aqueous:organic ratio. pH aqueous = 1.6 (25 °C)

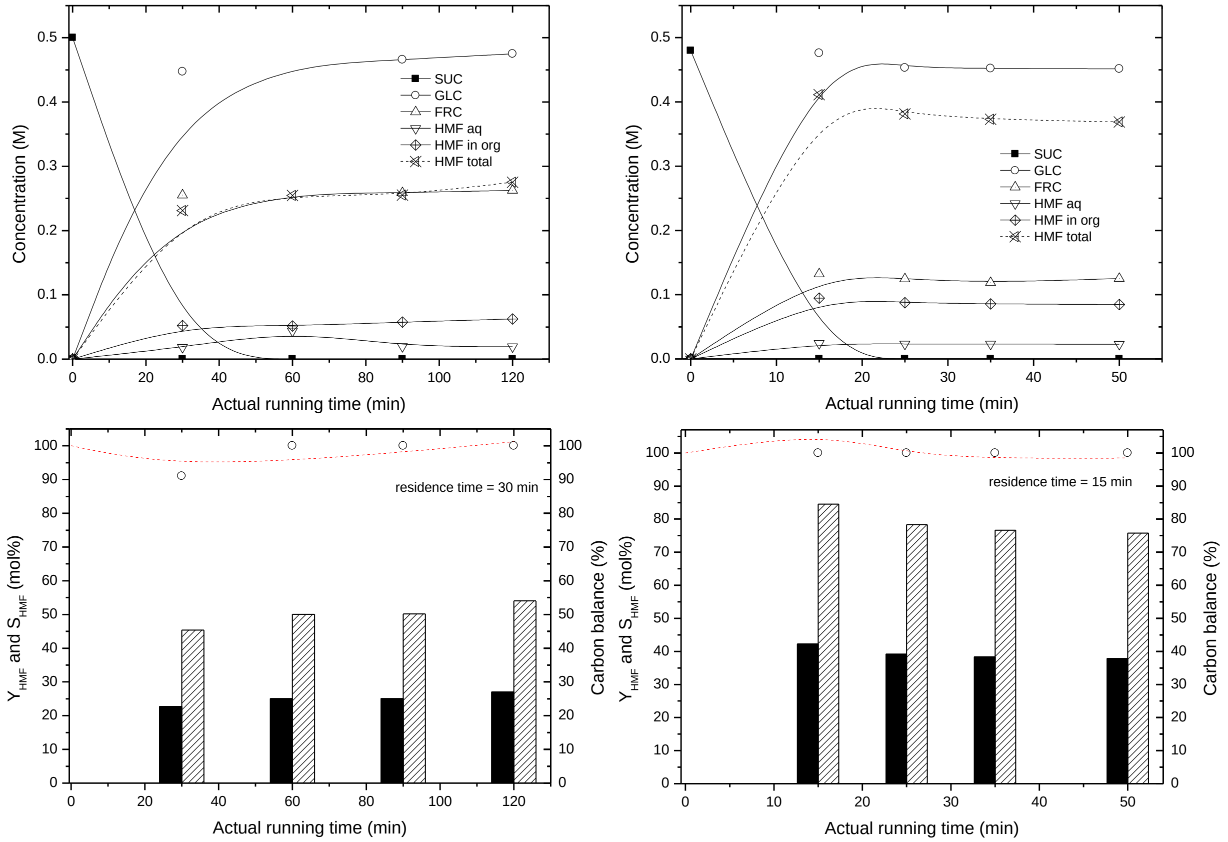

Figure S10. Concentration-time profile (above) and yield, selectivity and carbon balance (below) of thick juice hydrolysis in slug flow microreactor in biphasic system with MTHF extraction solvents, without addition of NaCl (left, 30 min residence time) and with addition of NaCl, 0.1 g/mL aqueous (right, 15 minutes residence time). Black bar: total HMF yield from SUC, shaded bar: HMF yield from FRC only, circle: HMF selectivity based on FRC only, red dash line. Reaction condition: $C_{SUC(equivalent),0}$ = 0.5 M, T = 150 °C, 1:4 v/v aqueous:organic ratio. $pH_{aqueous}$ = 1.2 (25 °C)

Table S4 Conversion of thick juice in microreactor at different running time. Reaction conditions: C_{SUC}=1 M, residence time 5 min, 150 ˚C, pH 1.2 (25 °C), 0.1 g/mL NaCl.

Running time	Y HMF, mol%	S HMF, mol%
5 min	14.8	38.0
10 min	23.6	53.3
15 min	53.8	89.3
10 h	55.1	92.5

Table S5 Conversion of thick juice in microreactor at different residence times at 1 M C_{suc}. Reaction 150 ˚C, pH 1.2 (25 °C), 0.1 g/mL NaCl.

Residence time	Y HMF, mol%	S HMF, mol%
5 min	55.1	92.5
10 min	79.3	93.3
15 min	89.8	91.3

Residence time and flowrate in microreactor

In the microreactor, the residence time and flowrate of the reaction mixture can be calculated as follows:

$$u_s = \frac{Q}{A_{tube}} \tag{S3}$$

$$\tau = \frac{L}{u_s} = \frac{L\,A_{tube}}{Q} \tag{S4}$$

Where:

u_s = superficial velocity of a given phase, m/min

Q = volume flow rate of the phase, m^3/min

A_{tube} = cross sectional area of the tube, m^2

τ = residence time, min

L = length of the reactor tube, m

The reaction in the microreactor was ran at 150 °C with MTHF acting as the extracting solvent. At this temperature, the volume of both aqueous and organic phases changes due to solubility of the two liquids and changes in the liquid density due to temperature effects. To compensate for this, the volume change of the different solvent ratios for water-MTHF system were also modeled in Aspen at 150°C (see Table S6) and the R value (for total volume change) was incorporated into equation S3, resulting in:

$$u_s = \frac{Q\,R_{tot}}{A_{tube}} \tag{S5}$$

Table S6. Modelled volume changes of aqueous:organic mixtures at 1:4 v/v ratio at various temperature as estimated by Aspen

For Water – MTHF mixture									
T (°C)	V_{aq} (L) Initial	V_{aq} (L) Final	R_{aq}	V_{org} (L) Initial	V_{org} (L) Final	R_{org}	V_{tot} (L) Initial	V_{tot} (L) Final	R_{tot}
140	100	48.50	0.4850	400	533.11	1.3328	500	581.61	1.1632
150	100	46.47	0.4647	400	545.18	1.3630	500	591.65	1.1833
160	100	43.55	0.4355	400	561.24	1.4031	500	604.79	1.2096

Example of microreactor flowrate calculation for a reaction run at 150 °C is as follows:

Reaction condition:

Reactor length = 4.5 m

Reactor i.d. = 1.651 mm

Desired residence time (τ) = 20 min

The superficial velocity of the mixture and residence time at 150 °C are calculated from:

$$u_s = \frac{L}{\tau} = \frac{4.5}{20} = 0.225\, m/min \tag{S6}$$

With correction on volume changes during reaction, the flowrate can be calculated as follow:

$$u_s = \frac{Q\, R_{tot}}{A_{tube}} = 0.225 \left(\frac{m}{min}\right) = \frac{Q * 1.1833}{\pi * \frac{0.001651^2}{4}} \left(\frac{1}{m^2}\right) \tag{S7}$$

$$Q = 4.07x10^{-7} \left(\frac{m^3}{min}\right) = 0.407\, ml/min \tag{S8}$$

To obtain residence time of 20 min, the HPLC pump was set at 0.407 mL/min flowrate. The HPLC pump used in this study has two inlet – one for organic and one for aqueous. Therefore, 0.407 mL/min was the total flowrate of both phases. The pump inlet setting was set to 20% for aqueous and 80% for organic to obtain the 1:4 v/v ratio of aqueous to organic phase. The complete residence time and flowrate used in this study are given in Table S7.

Table S7. Residence time and flowrate setting used in the continuous reaction with the slug-flow microreactor with MTHF as extracting solvent

Residence time (min)	(Total) Flowrate (mL/min)
5	1.628
10	0.814
15	0.523
20	0.407

References

[1] S. Lima, M.M. Antunes, A. Fernandes, M. Pillinger, M.F. Ribeiro and A.A. Valente. Acid-catalysed conversion of saccharides into furanic aldehydes in the presence of three-dimensional mesoporous Al-TUD-1, Molecules, 15 (2010), pp.3863-3877.

[2] J.N. Chheda, Y. Román-Leshkov and J.A. Dumesic. Production of 5-hydroxymethylfurfural and furfural by dehydration of biomass-derived mono-and poly-saccharides, Green Chemistry, 9 (2007) , pp.342-350.

[3] C. Moreau, R. Durand, C. Pourcheron and S. Razigade. Preparation of 5-hydroxymethylfurfural from fructose and precursors over H-form zeolites, Industrial Crops and Products, 3 (1994) , pp.85-90.

[4] Q.P. Peniston (Ed.), Manufacture of 5-hydroxymethyl 2-furfural1956.

[5] L. Rigal and A. Gaset. Direct preparation of 5-hydroxymethyl-2-furancarboxaldehyde from polyholosides: a chemical valorisation of the Jerusalem artichoke (Helianthus tuberosus L.), Biomass, 3 (1983) , pp.151-163.

[6] C.V. McNeff, D.T. Nowlan, L.C. McNeff, B. Yan and R.L. Fedie. Continuous production of 5-hydroxymethylfurfural from simple and complex carbohydrates, Applied Catalysis A: General, 384 (2010) , pp.65-69.

[7] S. Lima, P. Neves, M.M. Antunes, M. Pillinger, N. Ignatyev and A.A. Valente. Conversion of mono/di/polysaccharides into furan compounds using 1-alkyl-3-methylimidazolium ionic liquids, Applied Catalysis A: General, 363 (2009) , pp.93-99.

[8] B. Saha, S. De and M. Fan, Fuel 111 (2013)598-605.

[9] A. Jain, A.M. Shore, S.C. Jonnalagadda, K.V. Ramanujachary and A. Mugweru, Applied Catalysis A: General 489 (2015)72-76.

[10] Y. Shen, J. Sun, Y. Yi, M. Li, B. Wang, F. Xu and R. Sun, Bioresource Technology 172 (2014)457-460.

[11] G.R. Gomes, D.S. Rampon and L.P. Ramos, Applied Catalysis A: General 545 (2017)127-133.

[12] Q. Hou, W. Li, M. Zhen, L. Liu, Y. Chen, Q. Yang, F. Huang, S. Zhang and M. Ju. An ionic liquid–organic solvent biphasic system for efficient production of 5-hydroxymethylfurfural from carbohydrates at high concentrations, RSC Advances, 7 (2017), pp.47288-47296.

[13] A.G. Demesa, A. Laari, E. Tirronen and I. Turunen, Chemical Engineering Research and Design93 (2015)531-540.

[14] H.D. Hoving, D.A. Rijke, G.M.C. Wagemans, R.F.M.J. Parton and K. Babic. International Patents: WO2014037560, Process for the separation of levulinic acid from biomass (2014).
